# Supplementary material for: Measuring neuronal avalanches to inform brain-computer interfaces
Source: iScience. 2023 Dec 14;27(1):108734. doi: 10.1016/j.isci.2023.108734 (PMC10788504; doi:10.1016/j.isci.2023.108734)
Supplement: Document S1. Figures S1–S9, Tables S1, and S2 [file mmc1.pdf]

iScience, Volume *27*

Supplemental information

Measuring neuronal avalanches

to inform brain-computer interfaces

Marie-Constance Corsi, Pierpaolo Sorrentino, Denis Schwartz, Nathalie George, Leonardo L. Gollo, Sylvain Chevallier, Laurent Hugueville, Ari E. Kahn, Sophie Dupont, Danielle S. Bassett, Viktor Jirsa, and Fabrizio De Vico Fallani

Supplementary materials

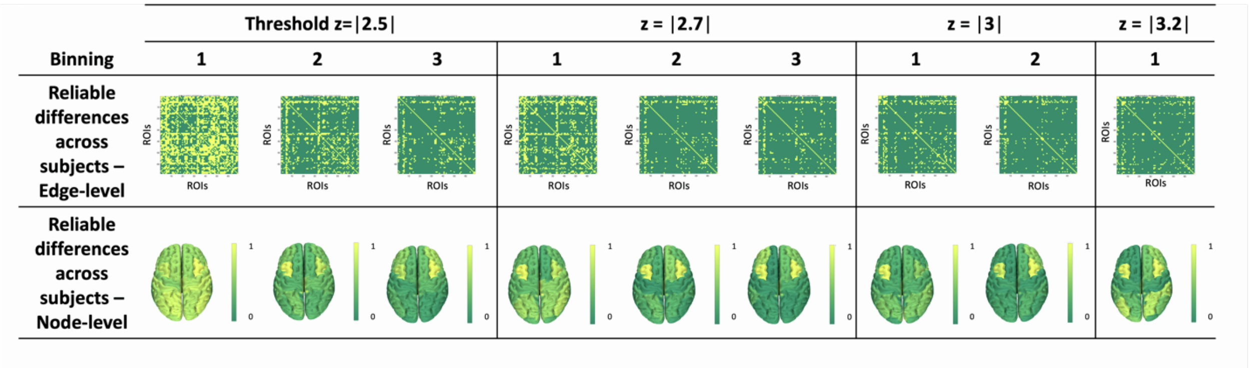

Figure S1. Replication analysis across thresholds and binnings, related to Figure 2.

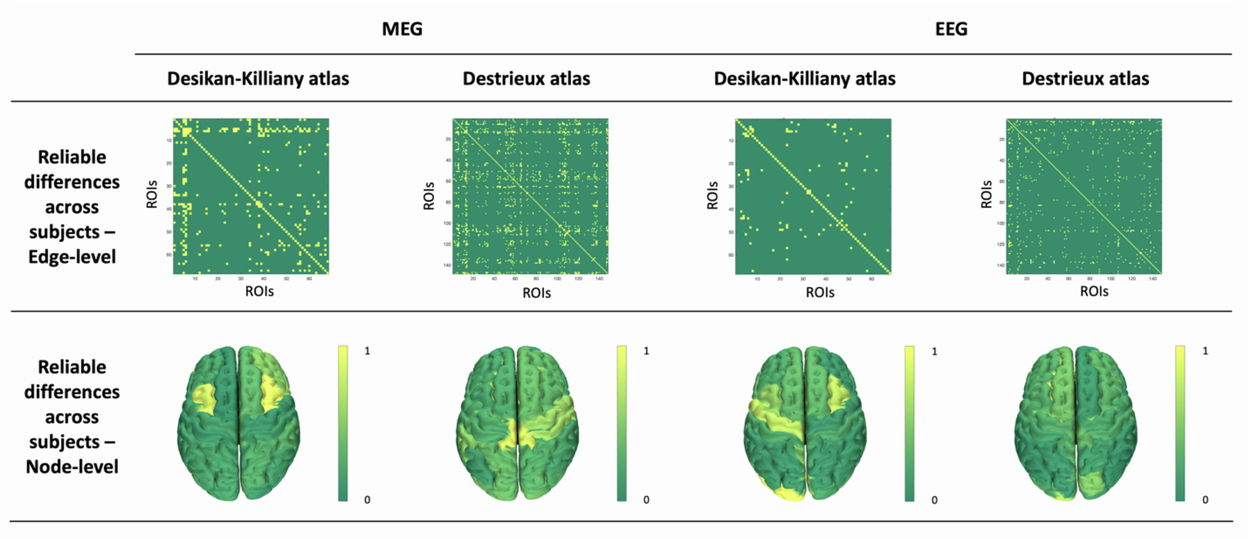

Figure S2. Replication analysis using EEG and the Destrieux atlas, related to Figure 2.

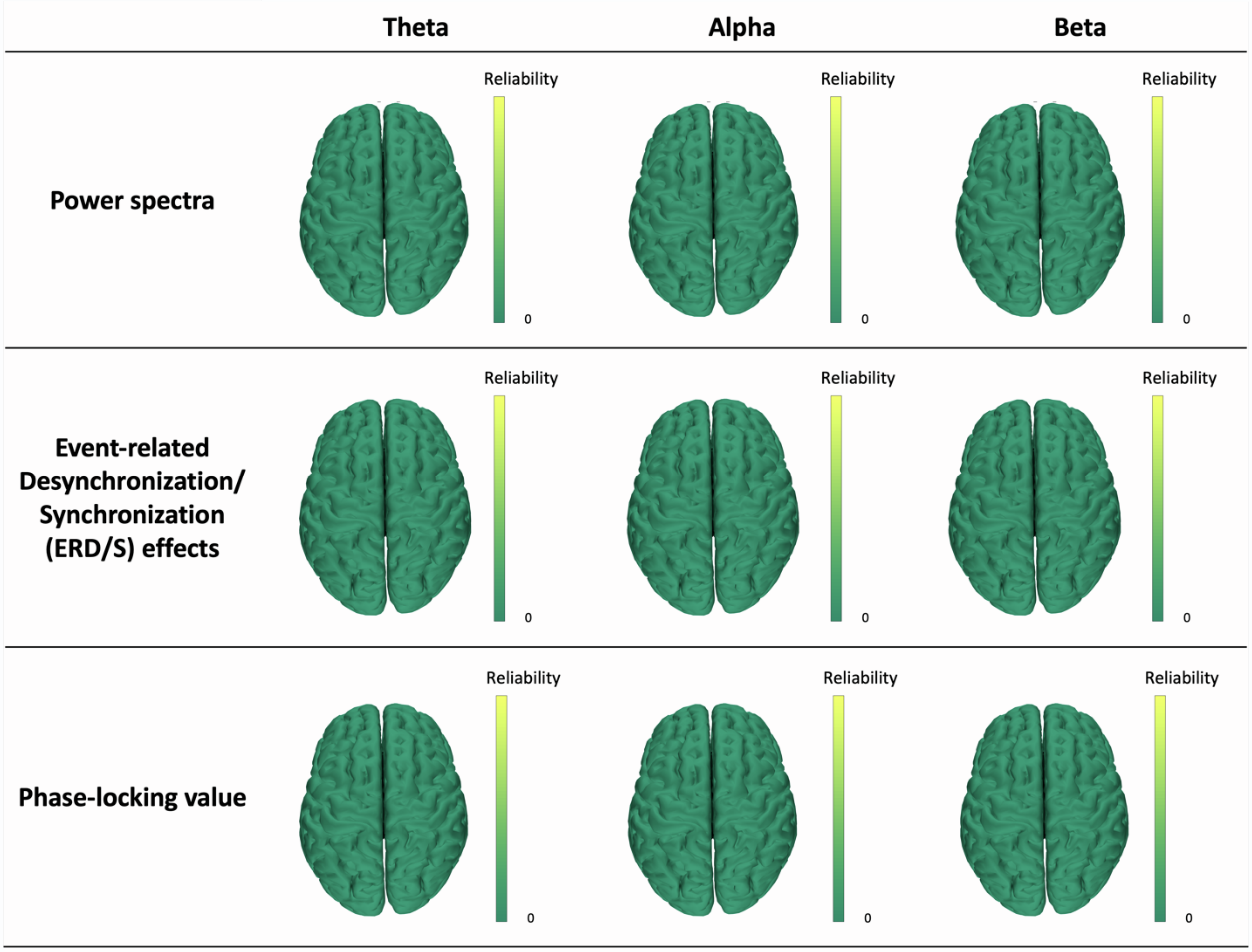

Figure S3. Reliability analysis performed on MEG data on features extracted respectively via power spectra, event-related desynchronization/synchronization (ERD/S) effects, and phase-locking value estimators, related to Figure 2.

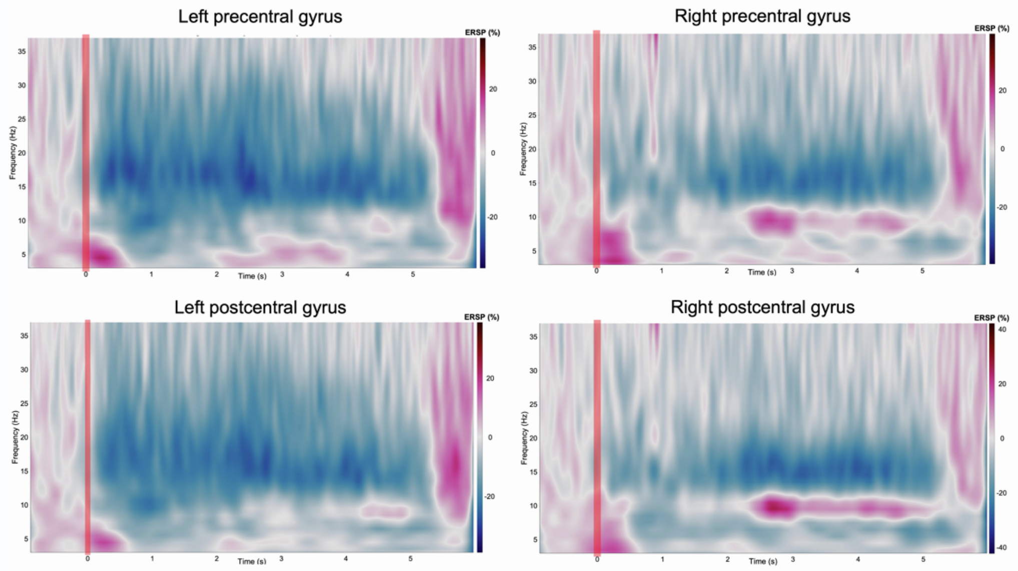

Figure S4. Grand average time-frequency analysis in Motor Imagery condition (n=20, MEG, Desikan-Kiliany) with ERD/S within the left and the right precentral gyri (first line) and the left and the right postcentral gyri (second line), related to Figure 2. t=0s corresponds to the moment when the target is displayed on the screen. t=5s corresponds to the moment when the result (ie hit/miss) is provided to the subjects.

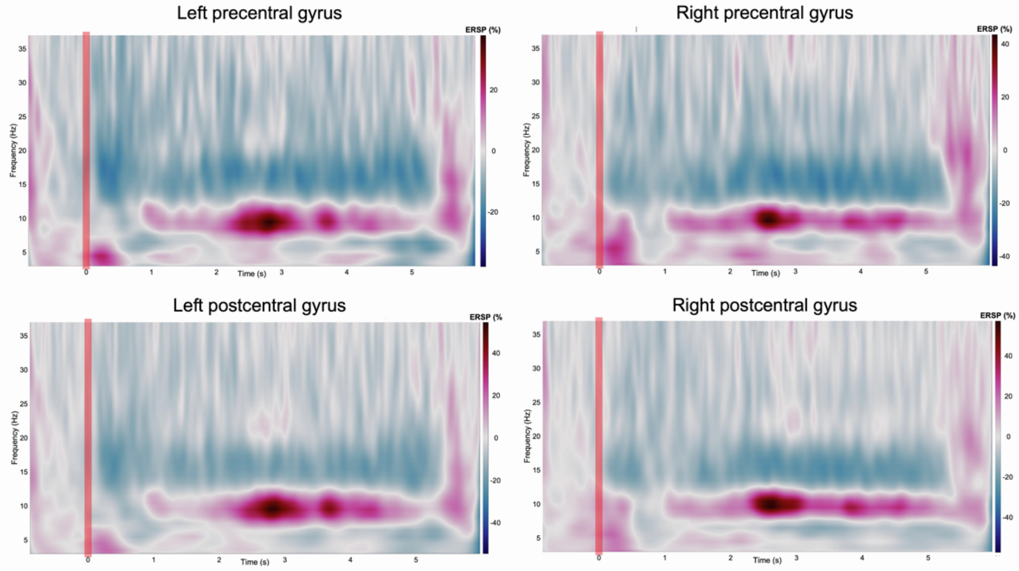

Figure S5. Grand average time-frequency analysis in the Rest condition (n=20, MEG, Desikan-Kiliany) with ERD/S within the left and the right precentral gyri (first line) and the left and the right postcentral gyri (second line), related to Figure 2. t=0s corresponds to the moment when the target is displayed on the screen. t=5s corresponds to the moment when the result (ie hit/miss) is provided to the subjects.

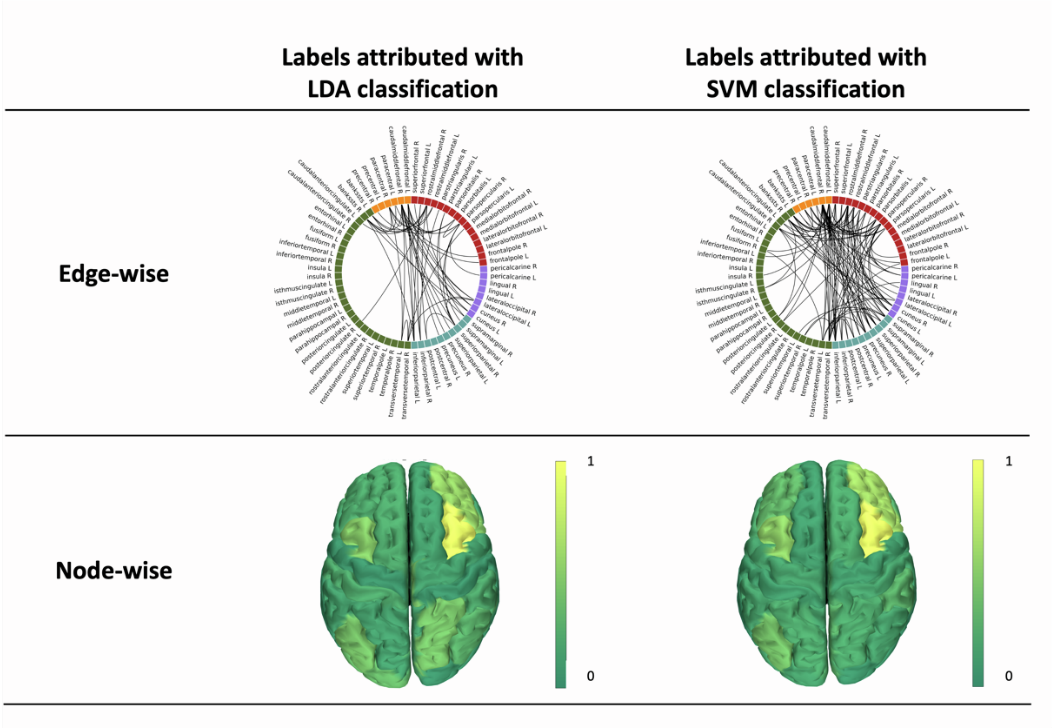

Figure S6. Influence of the classification tool on the reliability patterns edge and node-wise ($p<0.05$, BH corrected), related to Figure 2. On the left, the differences are derived from trial classification based on linear discriminant analysis (LDA), while on the right, the differences are derived from trial classification based on support vector machine (SVM).

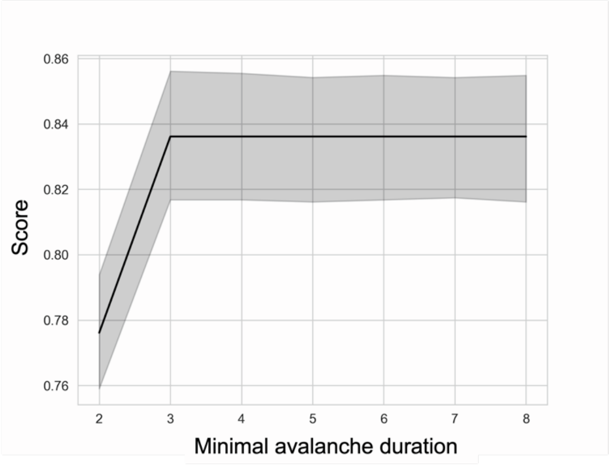

Figure S7. Influence of the minimal duration of the avalanches used to compute the ATM on the classification performance (with $|z| = 3$), related to Figure 3.

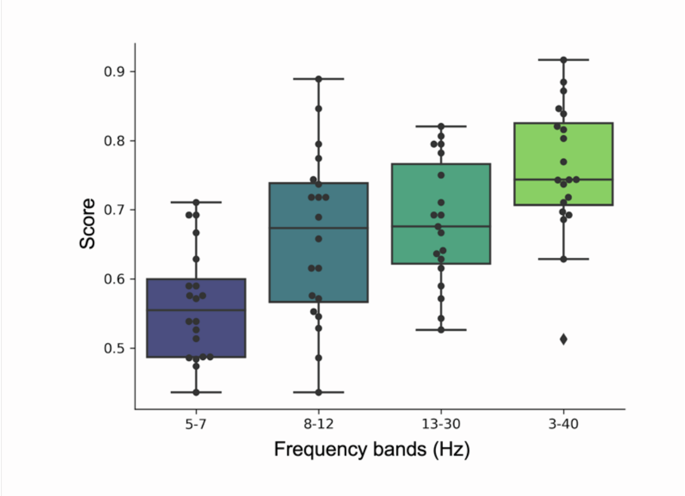

Figure S8. Influence of the frequency band on the classification performance with ATMs, related to Figure 3. For each frequency band, we plotted the distribution of the individual performance. Each dot represents the median of the scores obtained over the splits for each subject.

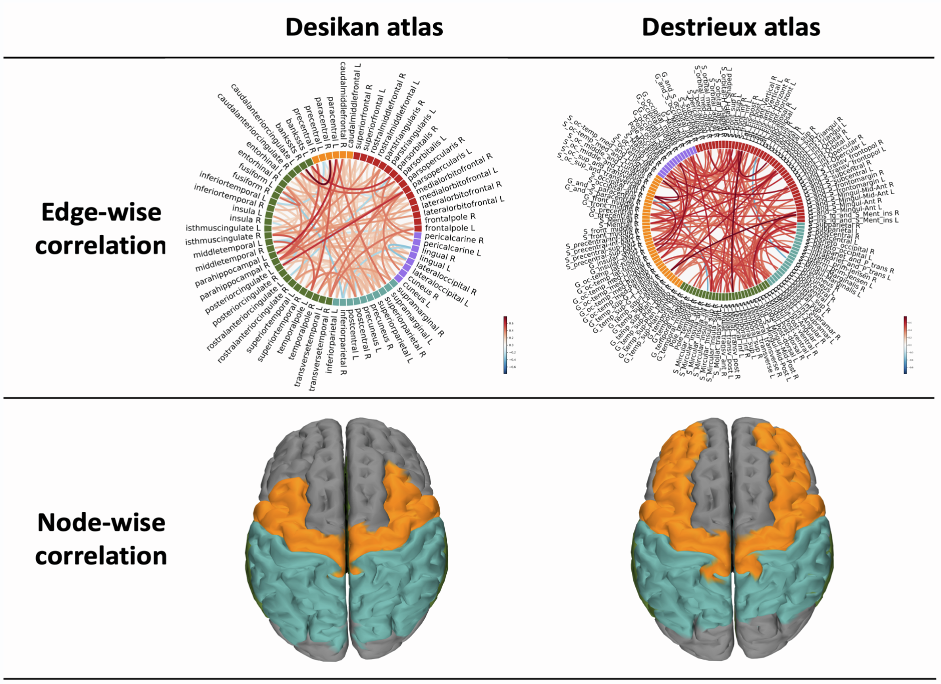

Figure S9. Replication analysis on MEG data using the Desikan and the Destrieux atlases, related to Figure 4. For visualization purposes, only the edges with |r| > 0.6 are visualized. The color of the edges is proportional to the correlation coefficient.

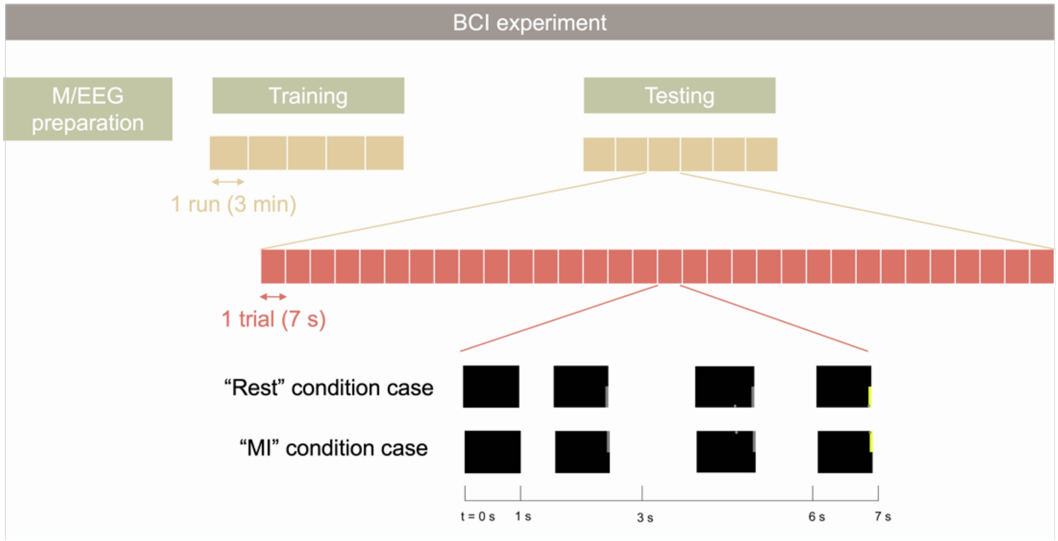

Figure S10. Online BCI experiment, related to STAR Methods.

Table S1. Participants characteristics, related to STAR Methods.

#Subject	Age	Gender (Male/Female)	BCI Score (%)
1	19	M	89.3
2	30	M	38.1
3	29	M	86.1
4	31	M	73.1
5	33	M	63.9
6	26	M	88.9
7	27	F	56.7
8	27	F	70.0
9	22	F	60.0
10	27	F	66.1
11	35	F	63.3
12	29	F	53.9
13	26	M	77.2
14	33	F	69.2
15	22	M	80.0
16	30	F	61.1
17	27	M	71.0
18	28	M	73.0
19	28	M	68.3
20	23	M	56.7

Table S2. List of regions of interests used in the study associated respectively with the Desikan-Killiany and the Destrieux atlases, related to STAR Methods.

#ROI	Desikan-Killiany atlas	Destrieux atlas
#1	bankssts L	G_Ins_lg_and_S_cent_ins L
#2	bankssts R	G_Ins_lg_and_S_cent_ins R
#3	caudalanteriorcingulate L	G_and_S_cingul-Ant L
#4	caudalanteriorcingulate R	G_and_S_cingul-Ant R
#5	caudalmiddlefrontal L	G_and_S_cingul-Mid-Ant L
#6	caudalmiddlefrontal R	G_and_S_cingul-Mid-Ant R
#7	cuneus L	G_and_S_cingul-Mid-Post L
#8	cuneus R	G_and_S_cingul-Mid-Post R
#9	entorhinal L	G_and_S_frontomargin L
#10	entorhinal R	G_and_S_frontomargin R
#11	frontalpole L	G_and_S_occipital_inf L
#12	frontalpole R	G_and_S_occipital_inf R
#13	fusiform L	G_and_S_paracentral L
#14	fusiform R	G_and_S_paracentral R
#15	inferiorparietal L	G_and_S_subcentral L
#16	inferiorparietal R	G_and_S_subcentral R
#17	inferiortemporal L	G_and_S_transv_frontopol L
#18	inferiortemporal R	G_and_S_transv_frontopol R
#19	insula L	G_cingul-Post-dorsal L
#20	insula R	G_cingul-Post-dorsal R
#21	isthmuscingulate L	G_cingul-Post-ventral L
#22	isthmuscingulate R	G_cingul-Post-ventral R
#23	lateraloccipital L	G_cuneus L
#24	lateraloccipital R	G_cuneus R
#25	lateralorbitofrontal L	G_front_inf-Opercular L
#26	lateralorbitofrontal R	G_front_inf-Opercular R
#27	lingual L	G_front_inf-Orbital L
#28	lingual R	G_front_inf-Orbital R
#29	medialorbitofrontal L	G_front_inf-Triangul L
#30	medialorbitofrontal R	G_front_inf-Triangul R
#31	middletemporal L	G_front_middle L
#32	middletemporal R	G_front_middle R
#33	paracentral L	G_front_sup L
#34	paracentral R	G_front_sup R
#35	parahippocampal L	G_insular_short L
#36	parahippocampal R	G_insular_short R
#37	parsopercularis L	G_oc-temp_lat-fusifor L
#38	parsopercularis R	G_oc-temp_lat-fusifor R
#39	parsorbitalis L	G_oc-temp_med-Lingual L
#40	parsorbitalis R	G_oc-temp_med-Lingual R
#41	parstriangularis L	G_oc-temp_med-Parahip L
#42	parstriangularis R	G_oc-temp_med-Parahip R
#43	pericalcarine L	G_occipital_middle L
#44	pericalcarine R	G_occipital_middle R
#45	postcentral L	G_occipital_sup L
#46	postcentral R	G_occipital_sup R
#47	posteriorcingulate L	G_orbital L
#48	posteriorcingulate R	G_orbital R
#49	precentral L	G_pariet_inf-Angular L
#50	precentral R	G_pariet_inf-Angular R
#51	precuneus L	G_pariet_inf-Supramar L
#52	precuneus R	G_pariet_inf-Supramar R

#53	rostralanteriorcingulate L	G_parietal_sup L
#54	rostralanteriorcingulate R	G_parietal_sup R
#55	rostralmiddlefrontal L	G_postcentral L
#56	rostralmiddlefrontal R	G_postcentral R
#57	superiorfrontal L	G_precentral L
#58	superiorfrontal R	G_precentral R
#59	superiorparietal L	G_precuneus L
#60	superiorparietal R	G_precuneus R
#61	superiortemporal L	G_rectus L
#62	superiortemporal R	G_rectus R
#63	supramarginal L	G_subcallosal L
#64	supramarginal R	G_subcallosal R
#65	temporalpole L	G_temp_sup-G_T_transv L
#66	temporalpole R	G_temp_sup-G_T_transv R
#67	transversetemporal L	G_temp_sup-Lateral L
#68	transversetemporal R	G_temp_sup-Lateral R
#69		G_temp_sup-Plan_polar L
#70		G_temp_sup-Plan_polar R
#71		G_temp_sup-Plan_tempo L
#72		G_temp_sup-Plan_tempo R
#73		G_temporal_inf L
#74		G_temporal_inf R
#75		G_temporal_middle L
#76		G_temporal_middle R
#77		Lat_Fis-ant-Horizont L
#78		Lat_Fis-ant-Horizont R
#79		Lat_Fis-ant-Vertical L
#80		Lat_Fis-ant-Vertical R
#81		Lat_Fis-post L
#82		Lat_Fis-post R
#83		Pole_occipital L
#84		Pole_occipital R
#85		Pole_temporal L
#86		Pole_temporal R
#87		S_calcarine L
#88		S_calcarine R
#89		S_central L
#90		S_central R
#91		S_cingul-Marginalis L
#92		S_cingul-Marginalis R
#93		S_circular_insula_ant L
#94		S_circular_insula_ant R
#95		S_circular_insula_inf L
#96		S_circular_insula_inf R
#97		S_circular_insula_sup L
#98		S_circular_insula_sup R
#99		S_collat_transv_ant L
#100		S_collat_transv_ant R
#101		S_collat_transv_post L
#102		S_collat_transv_post R
#103		S_front_inf L
#104		S_front_inf R
#105		S_front_middle L
#106		S_front_middle R
#107		S_front_sup L
#108		S_front_sup R

#109	S_interm_prim-Jensen L
#110	S_interm_prim-Jensen R
#111	S_intrapariet_and_P_trans L
#112	S_intrapariet_and_P_trans R
#113	S_oc-temp_lat L
#114	S_oc-temp_lat R
#115	S_oc-temp_med_and_Lingual L
#116	S_oc-temp_med_and_Lingual R
#117	S_oc_middle_and_Lunatus L
#118	S_oc_middle_and_Lunatus R
#119	S_oc_sup_and_transversal L
#120	S_oc_sup_and_transversal R
#121	S_occipital_ant L
#122	S_occipital_ant R
#123	S_orbital-H_Shaped L
#124	S_orbital-H_Shaped R
#125	S_orbital_lateral L
#126	S_orbital_lateral R
#127	S_orbital_med-olfact L
#128	S_orbital_med-olfact R
#129	S_parieto_occipital L
#130	S_parieto_occipital R
#131	S_pericallosal L
#132	S_pericallosal R
#133	S_postcentral L
#134	S_postcentral R
#135	S_precentral-inf-part L
#136	S_precentral-inf-part R
#137	S_precentral-sup-part L
#138	S_precentral-sup-part R
#139	S_suborbital L
#140	S_suborbital R
#141	S_subparietal L
#142	S_subparietal R
#143	S_temporal_inf L
#144	S_temporal_inf R
#145	S_temporal_sup L
#146	S_temporal_sup R
#147	S_temporal_transverse L
#148	S_temporal_transverse R

54
